# Knowledge about cervical cancer and barriers toward cervical cancer screening among HIV‐positive women attending public health centers in Addis Ababa city, Ethiopia

**DOI:** 10.1002/cam4.1334

**Published:** 2018-02-14

**Authors:** Saba Shiferaw, Adamu Addissie, Muluken Gizaw, Selamawit Hirpa, Wondimu Ayele, Sefonias Getachew, Eva J. Kantelhardt, Mathewos Assefa, Ahmedin Jemal

**Affiliations:** ^1^ Meshualekia Health Center Addis Ababa Ethiopia; ^2^ College of Health Sciences School of Public Health Addis Ababa University Addis Ababa Ethiopia; ^3^ Department of Gynecology Institute of Clinical Epidemiology Martin Luther University Halle and der Saale Germany; ^4^ Radiotherapy Center School of Medicine Addis Ababa University Addis Ababa Ethiopia; ^5^ American Cancer Society Atlanta Georgia

**Keywords:** Cervical Cancer, Screening, Ethiopia

## Abstract

Screening rate for cervical cancer among HIV‐infected women and among women overall is low in Ethiopia despite the high burden of the disease and HIV infection, which increases cervical cancer risk. In this paper, we assessed knowledge about cervical cancer symptoms, prevention, early detection, and treatment and barriers to screening among HIV‐positive women attending community health centers for HIV‐infection management in Addis Ababa. A cross‐sectional survey of 581 HIV‐positive women aged 21–64 years old attending 14 randomly selected community health centers without cervical cancer screening service in Addis Ababa. We used univariate analysis to calculate summary statistics for each variable considered in the analysis, binary logistic regression analysis to measure the degree of association between dependent and independent variables, and multiple regressions for covariate adjusted associations. Statistical significance for all tests was set at *P* < 0.05. We used thematic analysis to describe the qualitative data. Of the 581 women enrolled in the study with mean age 34.9 ± 7.7 years, 57.8% of participants had heard of cervical cancer and 23.4% were knowledgeable about the symptoms, prevention, early detection, and treatment of the disease. In multivariate analysis, higher educational attainment and employment were significantly associated with good knowledge about cervical cancer. In addition, only 10.8% of the participants ever had screening and 17% ever received recommendation for it. However, 86.2% of them were willing to be screened if free of cost. Knowledge about cervical cancer is poor and cervical cancer screening rate and provider recommendation are low among HIV‐positive women attending community health centers for management and follow‐up of their disease in Addis Ababa. These findings underscore the need to scale up health education about cervical cancer prevention and early detection among HIV‐positive women as well as among primary healthcare providers in the city.

## Introduction

HIV‐infected women have a fourfold excess risk of developing dysplasia and invasive cervical cancer largely because of reduced immunity 1, 2_._ Worldwide women with the highest risk of both HIV‐infection and cervical cancer are found in parts of Africa, including Ethiopia 3, 4. In Ethiopia, it is estimated that about 534,000 of women are HIV positive 5 and one in 30 females (0–74 years) develop cervical cancer in their lifetime 6.

Despite the high burden of cervical cancer 7 and HIV infection in Ethiopia 8, there is no large‐scale organized or opportunistic cervical cancer screening program, and cervical cancer screening rate among women is <1% 9. However, there is a small‐scale screen‐and‐treat cervical cancer screening program integrated with management of HIV infection in Addis Ababa (capital city) and a few other major cities, which is supported by Addis Tesfa (a local nonprofit organization) and Pathfinder International 5. Between 2010 and 2013, this program screened about 15,263 HIV‐positive women for cervical cancer in the whole country, although there were about 137,877 women living with HIV/AIDS in Addis Ababa alone 10. To mitigate this situation, Addis Tesfa and Pathfinder International, in collaboration with the Ethiopian government, are planning to expand cervical cancer screening programs to all government hospitals and health centers with HIV clinics in Addis Ababa and other major cities. However, the success of this initiative largely depends on health‐seeking behaviors and cervical cancer knowledge of HIV‐positive women in the community at large 11.

To date, few studies examined knowledge about cervical cancer and screening barriers among HIV‐positive women in Ethiopia and other parts of Africa where both the burden of cervical cancer and HIV infections are high 12, 13, 14. Further, these studies were limited to patients seen in a single or few select teaching or referral hospitals with screening services 14. Therefore, their finding may not be generalizable to HIV‐positive women treated in a community healthcare setting. In this study, we sought to assess knowledge about cervical cancer and identify barriers to screening among HIV‐positive women attending community health centers without screening services. We limited our analysis to health centers without screening services because they represent the majority of the centers.

## Methods

The study was conducted in public (community) health centers in Addis Ababa, the capital city of Ethiopia, with an estimated population of 3.35 million 15. There are 84 public health centers in the city, in addition to 51 government or private hospitals and around 700 private clinics. All public health centers initiate first‐line antiretroviral therapy (ART) treatments for uncomplicated patients and follow stable patients that have been transferred out from hospitals 16.

The study, conducted between February and March 2016, used facility‐based cross‐sectional study design with a mixed approach (both quantitative and qualitative). Sample size was determined for quantitative data using a single population proportion formula with following assumptions; proportion of knowledge about cervical cancer was taken as 31%, with 95% confidence interval (CI), and a 4% point margin of error for the estimate and 10% nonrespondent rate. The proportion on knowledge about cervical cancer was taken from a prior study done in northwestern part of Ethiopia where knowledge was defined as a mean score of pulled responses to a comprehensive list of knowledge questions on cervical cancer 17. This calculation resulted in a sample size of 594 participants. For the qualitative part, a total of 14 in‐depth interviews were conducted among five purposely selected adherence supporters (HIV‐positive women who provided counseling for HIV‐positive patients in the ART clinics concerning healthy living and adherence to treatment), five HIV‐positive patients and four healthcare providers working in ART clinics.

### Sampling procedures

From 84 public health centers found in Addis Ababa city administration, we first excluded 11 centers that integrated cervical cancer screening with ART service. Using a simple random sampling technique, we then selected 14 health centers from 73 of the remaining centers without screening services. The number of study participants to be included in each health center was determined in proportion with the total number of women who came to the ART and Pre‐ART services, using the patient flow 3 months prior to the data collection and at the time of data collection. Finally, systematic sampling technique was used to recruit the study participants. Of the total 594 HIV‐positive women aged 21–65 years invited to participate in the study, 581 women consented to participate in the study with 97.8% response rate.

### Data collection

Quantitative data were collected using a structured questionnaire. The questionnaire was first prepared in English and later was translated into the local language (*Amharic*). Pre‐testing of the questionnaire was carried out among 16 HIV‐positive women in health centers that were not included in the study. Data collectors, who were recruited from ART clinics for confidentiality purposes, were trained on data collection procedures.

For the qualitative part of data collection, we adapted a tool 18 that has been translated into *Amharic* and pretested for conducting an in‐depth interview of health‐seeking behavior for cervical cancer, including perceived barriers of cervical cancer screening among HIV‐positive women.

### Data analysis procedures

The data collection instruments were coded, and data were checked and entered using Epi‐info version 3.5.1 software (Centers for Disease Control (CDC), Atlanta, GA, USA). Data were cleaned and edited accordingly, checked for missing values and exported to SPSS version 20.0 statistical package for analysis (IBM Corporation, North Castle, New York, U.S.A.). First, the univariate analysis was used to describe frequency distribution, proportion, measures of central tendency, and dispersion. Then, binary logistic regression analysis was used to measure the association between the dependent variable and independent variables using the odds ratios and 95% CIs. Explanatory variables with *P*‐value of <0.2 in the bivariate analysis were included in the multiple logistic regression model. Statistical significance for the multiple logistic regression analysis was set at *α *≤ 0.05.

Knowledge of cervical cancer was assessed using 17‐point knowledge score. The respondents were asked a total of 17 multiple choice questions on knowledge that carried a total of 31 correct responses. Each correct response was given a score of 1 and wrong responses a score of 0. The points (questionnaires) included symptoms, prevention, early detection and treatment of the disease. Women with summary score greater than or equal to the mean value were categorized as having “good knowledge” and those with score less than the mean were categorized as having “poor knowledge”^17^. The mean was used to dichotomize the data for the logistic regression model. For the qualitative study, each in‐depth interview was tape‐recorded, transcribed and then translated. Data were coded and categorized according to ATLAS.ti software (ATLAS.ti Scientific Software Development GmbH, Berlin, Germany), and thematic analysis was used to describe the findings.

The study was approved by the Research Ethics Committee of the School of Public Health at College of Health Sciences of Addis Ababa University.

## Results

### Sociodemographic characteristics of the study participants

A total of 594 HIV‐positive women 21–65 years of age were targeted for the study, of whom 581 (97.8%) consented to participate in the study. The mean age and median household income of the study participants were 35 years (SD 7.7) and ETH 1000 birr (IQR 1300 birr), respectively; 22.2 birr was equivalent to one US dollar (Table 1).

**Table 1:** Sociodemographic characteristics of HIV‐positive women in Addis Ababa health centers, February 2016

Variables	Frequency (%)
Age category	
21–30	207 (35.6)
31–40	259 (44.6)
>40	115 (19.8)
Religion	
Orthodox	458 (78.8)
Protestant	72 (12.4)
Catholic	4 (0.7)
Muslim	47 (8.1)
Educational status	
Illiterates	150 (25.8)
Primary school	198 (34.1)
Secondary school and above	233 (40.1)
Occupational status	
Governmental/NGO employee	243 (41.8)
Self‐employed	167 (28.7)
Unemployed	171 (29.4)
Marital status	
Single	133 (23)
Married	239 (41)
Divorced/widowed/separated	209 (36)
Household income per month	
<700	159 (27.4)
700–2000	309 (53.2)
>2000	113 (19.4)
Duration of HIV diagnosis (years)	
<4	235 (40.4)
4–8	219 (37.7)
8–12	107 (18.4)
>12	20 (3.4)
ART treatment	
On ART	541 (93.1)
Not on ART	40 (6.9)
Know someone with cervical cancer	
Yes	61 (10.5)
No	520 (89.5)

About 239 of the study participants were married, and Orthodox Christianity dominated religion (*N* = 458, 79%). Participants with primary education and who were unemployed made up the largest proportion, 198 (34.1%) and 160 (27.5%), respectively. About 55% (323) of the study participants had 1–2 children; 61 (10.5%) of them knew someone with cervical cancer (Table 1).

Of the total 581 study participants, 57.8% of them had heard of cervical cancer; the most common source of information was mass media (65.9%), followed by healthcare providers (20.1%). One hundred eighty‐six participants (32% of total) had heard of cervical cancer screening, again with the media (52.7%) and healthcare providers (40.8%) as the two major sources of information. Overall, only 23.4% of the study participants were knowledgeable about cervical cancer symptoms, prevention, screening and treatment.

All of the 14 in‐depth interview respondents had heard about cervical cancer, but the majority of them did not have detailed knowledge regarding cervical cancer, including the HIV‐positive women counselors and the healthcare providers working in the ART clinics.

### Knowledge of cervical cancer symptoms, prevention and treatment methods

About 44% (147) of the study participants mentioned foul vaginal discharge, 95 (28.3%) vaginal bleeding, 50 (14.9%) pelvic or back pain, and 49 (14.6%) pain during coitus as symptoms of cervical cancer, while 129 (38.4%) did not name any of the specific symptoms for the disease.

Concerning preventability of cervical cancer, 294 (50.6%) of participants believe that cervical cancer is preventable. Of these, 95 (32.3%) participants believed cervical cancer can be prevented through screening and 27 (9.2%) through vaccination; 375 (64.5%) of the study participants responded that cervical cancer is treatable if detected early. Radiation was identified as a treatment method by 110 (18.9%) study participants, while 390 (67.1%) did not know of any treatment modalities for cervical cancer (Table 2).

**Table 2:** Knowledge of HIV‐positive women about prevention and treatment mechanisms of cervical cancer in Addis Ababa health centers, February 2016

Variable	Frequency	Percentage	95% CI
Good knowledge	136	23.4	0.19–0.26
Poor knowledge	445	76.6	0.73–0.8
Having multiple sexual partner is a risk of	164	48.8	0.46–0.51
Exposure to sexual debut is a risk of cervical cancer	88	26.2	0.21–0.3
Infection by virus (HPV) is a risk of cervical cancer	40	11.9	0.08–0.15
Low immunity due to HIV/AIDS is a risk of cervical cancer	21	6.3	0.04–0.08
Vaginal discharge is a symptom of cervical cancer	147	43.8	0.38–0.49
Vaginal bleeding is a symptom of cervical cancer	95	28.3	0.23–0.33
Pain during coitus is a symptom of cervical cancer	49	15	0.11–0.18
Cervical cancer is preventable	294	50.6	0.46–0.54
Vaccination prevents cervical cancer	27	9.2	0.06–0.12
Screening prevents cervical cancer	95	32.3	0.26–0.37
Cervical cancer screening age for HIV +ve women is as soon as she became positive	111	19.1	0.16–022
Cervical cancer screening age for HIV‐positive women is at any age	68	11.7	0.092–0.14
Cervical cancer screening frequency for HIV +ve women is every 3 years	37	6.4	0.04–0.08
Cervical cancer screening frequency for HIV +ve women is every 5 years	17	2.9	0.02–0.035
Don't know the screening frequency	212	36.5	0.32–0.4
Cervical cancer is treatable disease	375	64.5	0.6–0.68
Surgery is a treatment modality for cervical cancer	60	10.3	0.08–0.12
Radiation is a treatment modality for cervical cancer	110	18.9	0.16–0.22
Chemotherapy is a treatment modality for cervical cancer	53	9.1	0.067–0.11
Don't know the treatment for cervical cancer screening	390	67.1	0.63–0.7

Good knowledge, knowledge score of more than the mean value; Poor knowledge, knowledge score of less than the mean value.

The mean value of the knowledge is calculated by considering the responses of all study participants for the 17 knowledge‐based questions 17.

#### Qualitative study findings

Most healthcare provider respondents mentioned foul smelling vaginal discharge as a symptom for cervical cancer, whereas none of HIV‐positive women mentioned at least one cardinal cervical cancer symptom.

Majority of HIV‐positive respondents reported that cervical cancer can be prevented by avoiding multiple sexual partners and by using condom. But none of them believed that it can be successfully treated once it is diagnosed.

A 40‐year‐old HIV‐positive woman said

cervical cancer is preventable by taking care of yourself; for example, by practicing personal hygiene. You also need to have a faithful relationship with your partner, as unfaithful men might transmit the disease to you.

### Knowledge on recommended screening age and frequency for cervical cancer

A total of 111 study respondents (19.1%) said the recommended age for HIV‐positive women to begin cervical cancer screening is as soon as she becomes HIV positive; 191 (32.9%) did not know this (Table 2). The most frequently mentioned time interval for cervical cancer screening for HIV‐positive women was every year 169 (29.1%), while 212 (36.5%) of the participants did not have any idea about the frequency of screening. Findings from the in‐depth interviews indicated that none of the interviewed respondents including healthcare providers working in an ART clinic correctly identified the recommended age and frequency of cervical cancer screening for HIV‐positive women (Table 2).

### Attitude and practice of cervical cancer screening

As shown in Table 2, 94.1% (547) of study participants believe that early detection of cervical cancer is important. Of the study participants, 481 (82.8%) responded that no one recommended them to undergo cervical cancer screening. Further, 498 (85.7%) reported being willing to undergo cervical cancer screening if the screening service was free and integrated to the ART clinic. Findings from the in‐depth interviews indicated that most of the women would undergo screening if the screening service was at nearby health centers or integrated into the ART clinic.

Of the total study participants, 63 (10.8%) had been screened for cervical cancer at least once in their lifetime. Among those screened, 49 (77.8) had been screened once, 12 (19%) had been screened twice, and 2 (3.2%) has been screened three times; 58 (93.5%) were willing to be screened again. Of the five participants who were unwilling to be screened again, three reasoned that the screening procedure is painful (Table 3).

**Table 3:** Attitude and practice of HIV‐positive women toward cervical cancer screening in Addis Ababa health centers, February 2016

Variable	Frequency	Percentage	95% CI
It is important to detect cervical cancer at an early stage	547	94.1	0.92–0.95
Someone recommended me to practice screening	99	17	0.1–0.24
I am willing to undergo pelvic examination	495	85.2	0.82–0.88
I am willing to undergo cervical cancer screening	491	84.5	0.8–0.87
I am willing to practice screening if it is integrated to ART clinic	498	85.7	0.82–0.88
I am willing to practice screening in my catchment health center	485	83.5	0.8–0.86
I am willing to practice screening with payment	324	55.8	0.5–0.61
Have you ever been screened?			
Yes	63	10.8	0.02–0.17
No	518	89.2	0.87–0.9
How many times?			
One times	49	77.8	0.66–0.88
Two times	12	19	0.03–0.4
Three times	2	3.2	0.2–0.27
When was the last time you were screened?			
Within the last 3 years	47	74.6	0.62–0.86
Within the last 5 years	7	11.1	0.12–0.34
Before 5 years	9	14.3	0.08–0.36
Would you like to be screened again?			
Yes	59	93.5	0.86–0.99
No	4	6.5	0.173–0.3

### Perceived barriers toward cervical cancer screening

The most frequently mentioned barrier toward screening by study participants was that they were feeling healthy (*n* = 212, 36.5%). Lack of knowledge about cervical cancer and cervical cancer screening, perceived fear of positive results, perceived pain during screening procedure and financial factors were the most common barriers mentioned by in‐depth interview respondents (Table 4).

**Table 4:** Reasons given for not attending cervical cancer screening among HIV‐positive women in Addis Ababa health centers, February 2016

Variables	Frequency	Percentage
Individual factors		
I am healthy	212	36.5
Never think of	139	23.9
I don't know where screening is done	117	20.1
Lack of awareness about screening	56	9.6
Embarrassing	32	5.5
Fear of positive results	31	5.3
Painful	7	1.2
I don't know about cervical cancer	6	1
Social factors		
Partner attitude	8	1.4
Religion factors	5	0.9
Health care‐related factors		
No appropriate care at HCF	36	6.2
No health facility in the catchment area	25	4.3
HCP do not have good knowledge	8	1.4
HCP attitude	4	0.7
Financial‐related factors		
It is expensive	33	5.7
Others	5	0.9

### Association between sociodemographic characteristics and knowledge score

In binary logistic regression analysis, primary education (OR 95% CI, 2.32 (1.25–4.3)) as well as secondary education and above (OR 95% CI, 4.1 (2.3–7.4)), self, government or NGO employment g (OR 95% CI, 2.7 (1.63–4.4)), higher household income (OR 95% CI, 2.8 (1.58–4.95)) and longer duration since HIV diagnosis (OR 95% CI, (2.8 (1.1–7.1)) were associated with good knowledge of cervical cancer and cervical cancer screening. However, the association remained statistically significant only for primary (OR 95% CI, 2.0 (1.08–4)) as well as secondary and higher (OR 95% CI, 2.94 (1.5–5.6)) educational attainment and for employment status (OR 95% CI, 2.0 (1.2–3.48)) when adjusted for potential confounders in the multiple logistic regression analysis (Table 5).

**Table 5:** Association of sociodemographic characteristics and knowledge score in Addis Ababa health centers, February 2016

Variables	Good knowledge	Poor knowledge	COR (95%_CI)	AOR (95%_CI)
Educational status				
Illiterates	16 (10.6%)	134 (89.3%)	1	1
Primary school	43 (21.7%)	155 (78.3%)	2.32 (1.25–4.3)	2.0 (1.08–4)
Secondary school and above	77 (33%)	156 (67%)	4.1 (2.3–7.4)	2.94 (1.5–5.6)
Occupational status				
Government/NGO employee	77 (31.7%)	166 (68.3%)	2.7 (1.63–4.4)	2.0 (1.2–3.48)
Self‐employed	34 (20.4%)	133 (79.6%)	1.5 (0.88–2.8)	1.6 (0.9–2.9)
Unemployed	25 (14.6%)	146 (85.4%)	1	1
Household income				
<700	26 (16.4%)	133 (83.6%)	1	1
700–2000	70 (22.7%)	239 (77.3%)	1.49 (0.9–2.46)	1.05 (0.62–1.8)
>2000	40 (35.4%)	73 (64.6%)	2.8 (1.58–4.95)	1.6 (0.85–3.03)
Time since HIV diagnosis (years)				
<4	53 (22.6%)	182 (77.4%)	1	1
4–8	46 (21%)	173 (79%)	0.9 (0.58–1.42)	0.98 (0.61–1.56)
8–12	28 (26.2%)	79 (73.8%)	1.2 (0.71–2.1)	1.2 (0.72–2.1)
>12	9 (45%)	11 (55%)	2.8 (1.1–7.1)	2.6 (0.98–7)

COR (95%_CI), Crude odds ratio with 95% confidence interval; AOR (95%_CI), adjusted odds ratio with 95% confidence interval.

## Discussion

To our knowledge, this is the first large study to examine cervical cancer knowledge and barriers to cervical cancer screening among HIV‐positive women attending community health centers for HIV management in Addis Ababa. We found that a little more than half of the participants had heard of cervical cancer, a third heard of cervical cancer screening, a tenth ever had been screened, and nine in 10 never received screening recommendation from their healthcare providers although the majorities (86%) of the participants were willing to undergo screening if it was free of cost.

Knowledge is a prerequisite to practicing screening; however, women in many developing countries lack knowledge about cervical cancer and cervical cancer screening service 15, 19, 20. More than half of the participants in our study had heard about cervical cancer, which is comparable with findings among HIV‐positive women in Nigeria 21, 22 but significantly higher compared to findings in southern Ghana 23. In contrast, level of awareness of the disease is much higher among women in some African countries such as in Kinshasa, Republic of Congo (81.9%) and in Botswana (77%), and in female sex workers in China (70.2%) 19, 24, 25. These regional differences in part reflect differences in socio demographic characteristics of study participants and setting, that is, all women versus HIV‐positive women and health facility versus community‐based studies. Regardless, for screening methods to be fully utilized, women must first be made aware of the disease and the availability of screening methods 26.

Of those study participants who heard of cervical cancer, we found that the majority of them heard it via the media, and a few from healthcare personnel and friends and neighbors. This is similar to findings from a previous study in the northwest part of Ethiopia 17. In contrast, a study conducted among Kinshasa women showed that more than half of participants knew of cervical cancer via conversation with other people, and less frequently through media 24. Our finding of media as the major source of information for cervical cancer knowledge among HIV‐positive women in Addis Ababa may in part reflect the substantial media coverage of the recent government/civil society initiatives to expand cervical cancer screening in the city and other parts of the country.

A health officer who worked for 3 years in ART clinic said

We have weakness on this side, most of the time we don't tell them about screening unless they seek or we suspect the disease. I believe, we should do a better job in this area.

Half of 294 (50.6%) of the study participants said cervical cancer is preventable, which is substantially higher compared to findings in other settings. Much lower proportion was reported in Johannesburg, Burkina Faso, and LAO PDR 27, 28, 29. These variations may in part reflect differences in awareness creation strategies, including the extent of media coverage, and as well as cultural differences. Nonetheless, knowing that cervical cancer is preventable will motivate women to avoid risky behaviors as well to participate in screening.

We found less than one‐tenth and a third of the study participants mentioned vaccination and screening, respectively, as preventive measures for cervical cancer. These findings were comparable to those from Nigeria and Gabon 30, 31. According to a review of cancer screening in 57 countries, screening rate prevalence is <1% in Ethiopia, although screening all women in the target age group every 3 years is estimated to prevent 91% of cases 9. There is a national program promoting VIA screening for free. The Ethiopian government has also recently introduced HPV vaccination demonstration project. However, the HPV vaccination program is not yet available as a national program awaiting the results of the demonstration project. Vaccination against HPV types 16 and 18 (recommended ages 9–13 years) is effective in preventing chronic HPV infections, which cause over 70% of cervical cancer 32.

Although only a third of study participants knew cervical cancer can be prevented through screening; the majority of participants believe that cervical cancer is treatable if detected early. Much lower results were found in north central Nigeria and Ogun state in Nigeria 31, 33. In contrast, much higher results were reported among Chinese women (80.8%) 25. Currently in Ethiopia, many governmental and nongovernmental organizations have programs or campaigns to increase uptake of cervical cancer screening mainly using visual inspection with acetic acid (VIA) and treatment (cryotherapy) of abnormal lesions immediately following screening. For women who test positive, referral system has been in place for treatment with loop electrosurgical excision procedure for early‐stage lesions and other methods for advanced‐stage diseases 10, 11.

We found that only a tenth of study participants had ever been screened for cervical cancer, similar to study findings among HIV‐positive women in Addis Ababa and Nigeria 12, 31. However, 86% of the respondents in our study were willing to undergo screening and more than three‐fourths of the study participants had positive attitudes toward screening services integrated with HIV‐infection management. The lack of screening services in the community health centers may in part reflect the low uptake of screening in these women.

Finance is another perceived barrier to screening. Participants' willingness to undergo screening was much lower for services that require payment. This is unsurprising as most study participants were of low socioeconomic status. Similar findings on cost as a barrier to screening were reported among HIV‐positive women in Nigeria and Côte d'Ivoire 22, 34. In Ethiopia, cervical cancer screening services through VIA are provided for free in public health facilities. However, the free availability of services for free may not have been promoted consistently.

Additional barriers to cervical cancer screening identified in this study included feeling healthy and lack of provider recommendations for screening. One‐third of the respondents mentioned their perception of feeling healthy as a reason to not undergo cervical cancer screening. However, most women in Ethiopia and other parts of sub‐Saharan Africa often present to health facilities at advanced stages of the disease, having previously thought they were healthy 26. Further, only 17% of participants in our study ever received cervical cancer screening recommendations from their provider in view of provider recommendation is the singular most important factor for receipt of screening and other preventive and healthcare services 35.

The major barrier toward screening identified among in‐depth interviews was lack of awareness about cervical cancer. Some respondents also mentioned fear of the test results. A study conducted in LAO PDR had similar findings, in that the majority of the respondents reasoned no symptoms, followed by having never heard of screening 27. HIV‐positive South African and Nigerian women reported fear of procedure and anticipated cost of the test, respectively, as the main barrier 13, 22. Findings differed in southern Ghana, where lack of screening sites, distance to screening sites, limited information on cervical cancer, and absence of health education programs were their principal reasons for not practicing screening 23.

This study indicated that the odds of being knowledgeable about cervical cancer is lower among HIV‐positive women with lower level of education than among women with higher educational levels, similar to the LAO PDR study 27. This difference could be attributed to differences in disease severity perception by educational status, with women with higher level of education more likely to obtain information about cervical cancer via social media (internet and television). Similarly, this study also revealed that the odds of good knowledge about cervical cancer among government and nongovernmental organization employees were two times higher than among unemployed participants.

The strength of our study is the use of mixed methods for triangulating the quantitative findings by the qualitative findings. Data were collected via face‐to‐face interviews, minimizing the likelihood of misunderstanding the questions. Moreover, the study was conducted among randomly selected health centers in Addis Ababa, representing HIV‐positive women attending public health centers for routine management of their disease. However, the study excludes HIV‐positive women who receive HIV care from private healthcare institutions, and these women are more likely to have higher educational attainment and income than those HIV‐positive women attending public health centers. However, HIV‐positive women attending private hospitals or clinics are less likely to represent a small proportion of all HIV‐positive women in the city. In addition, although the data collectors were trained, interviewer bias cannot be excluded as they were selected from the ART clinics for purposes of confidentiality. The interpretation of our finding may also have been affected by social desirability bias 36.

## Conclusion and Recommendation

We found that knowledge about cervical cancer is poor among HIV‐positive women attending public health centers in Addis Ababa, Ethiopia, with less than a quarter of them being knowledgeable about the disease and a third of them could not mention any one of common cervical cancer symptoms. Further, only one in 10 participants ever screened for cervical cancer, although the majority of them were willing to undergo screening if free of cost. In addition to lack of patient knowledge of knowledge about the disease, lack of provider recommendations is a major barrier to cervical cancer screening in the city. These findings underscore the need to scale up health education about cervical cancer prevention and early detection among HIV‐positive women as well as among primary healthcare providers in Addis Ababa for successful expansion of cervical cancer screening in the city and perhaps in other parts of the country.

## Conflict of Interest

None declared.
